# Supplementary material for: The interactome of intact mitochondria by cross-linking mass spectrometry provides evidence for coexisting respiratory supercomplexes
Source: Mol Cell Proteomics. 2017 Dec 8;17(2):216–32. doi: 10.1074/mcp.RA117.000470 (PMC5795388; doi:10.1074/mcp.RA117.000470)
Supplement: Supplemental Data [file supp_RA117.000470_133922_0_supp_23978_fzffwf.zip › spectra_annotation/mito_spectra_annotation/76-2-11-1-11-1.pdf]

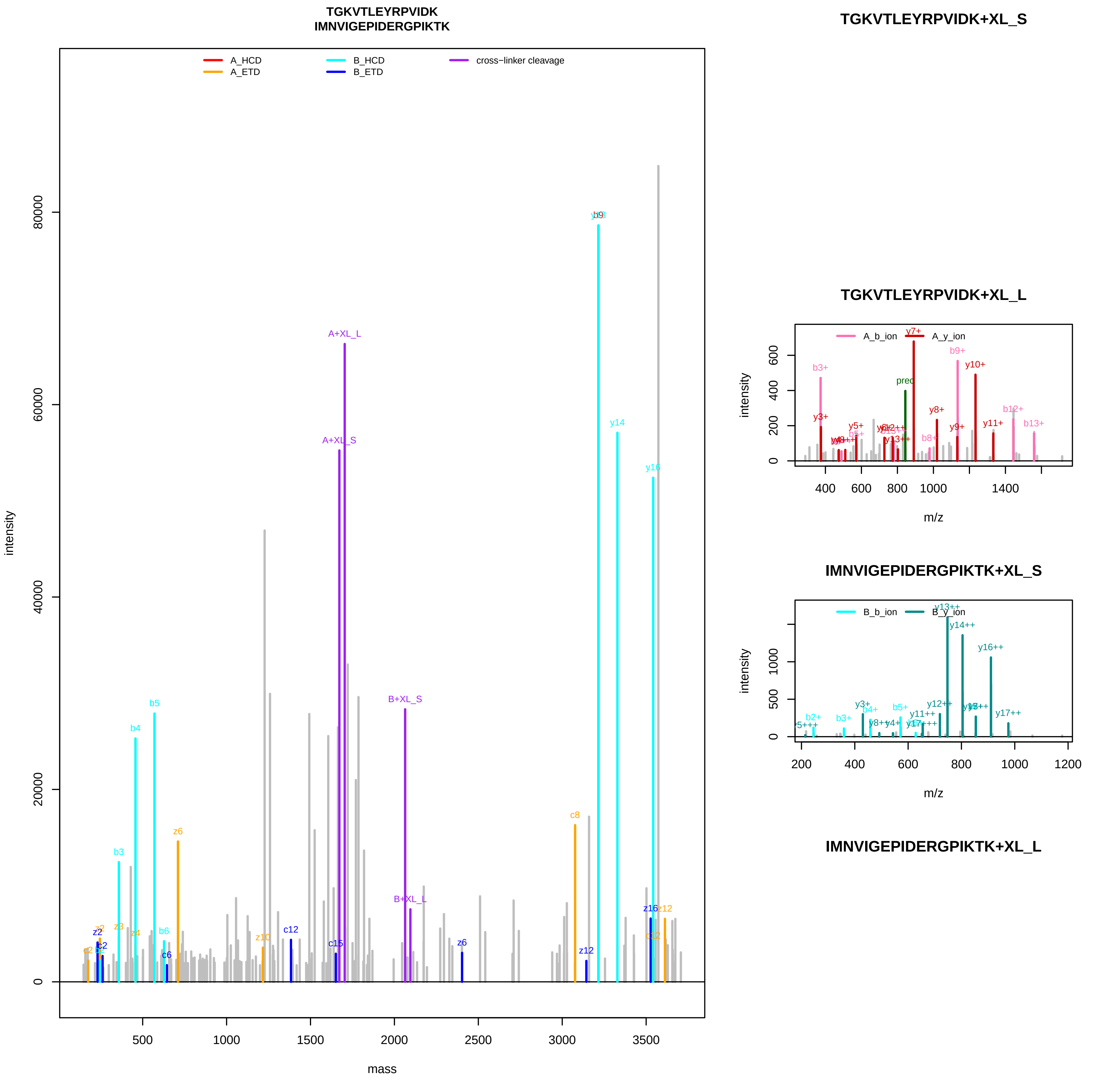
TGKVTLEYRPVIDK
IMNVIGEPIDERGPIKTK
A_HCD
A_ETD
B_HCD
B_ETD
cross−linker cleavage
intensity
mass
0
20000
40000
60000
80000
500
1000
1500
2000
2500
3000
3500
A+XL_L
A+XL_S
B+XL_S
B+XL_L
b3
b4
b5
b6
c6
c12
c15
c8
z6
z10
z12
z16
z6
y14
y16
TGKVTLEYRPVIDK+XL_S
TGKVTLEYRPVIDK+XL_L
A_b_ion
A_y_ion
intensity
m/z
400
600
800
1000
1400
b3+
y3+
y5+
y7+
b9+
y10+
y8+
y9+
y11+
b12+
b13+
b8+
prec
IMNVIGEPIDERGPIKTK+XL_S
B_b_ion
B_y_ion
intensity
m/z
200
400
600
800
1000
1200
y13++
y14++
y16++
y12++
y11++
y17++
b2+
b3+
y3+
b4+
b5+
IMNVIGEPIDERGPIKTK+XL_L
